# Development of a set of process and structure indicators for palliative care: the Europall project

**DOI:** 10.1186/1472-6963-12-381

**Published:** 2012-11-02

**Authors:** Kathrin Woitha, Karen Van Beek, Nisar Ahmed, Jeroen Hasselaar, Jean-Marc Mollard, Isabelle Colombet, Lukas Radbruch, Kris Vissers, Yvonne Engels

**Affiliations:** 1 Department of Anaesthesiology, Pain and Palliative Medicine, Radboud University Nijmegen Medical Centre, Geert Grote Plein 10, Nijmegen 6500 HB, The Netherlands; 2 Department of Radiotherapy-Oncology and Palliative Medicine, University Hospital Leuven, Leuven, Belgium; 3 Academic Unit of Supportive Care, School of Medicine and Biomedical Sciences, The University of Sheffield, Sykes House, Little Common Lane, Sheffield, S11 9NE, UK; 4 Department of Science and Research in Palliative Medicine, University of Bonn, Malteser Hospital Bonn/Rhein-Sieg, Bonn, Germany; 5 Réseau de Santé, Paris Sud, France; 6 Université Paris Descartes, Sorbonne Paris Cité, Public Health, Paris, F-75006, France; 7 AP-HP, Cochin Teaching Hospital, Palliative Medicine, Paris, F-75014, France

**Keywords:** Quality indicator, Organisation, Europe, Public health, Palliative care, Europall

## Abstract

**Background:**

By measuring the quality of the organisation of palliative care with process and structure quality indicators (QIs), patients, caregivers and policy makers are able to monitor to what extent recommendations are met, like those of the council of the WHO on palliative care and guidelines. This will support the implementation of public programmes, and will enable comparisons between organisations or countries.

**Methods:**

As no European set of indicators for the organisation of palliative care existed, such a set of QIs was developed. An update of a previous systematic review was made and extended with more databases and grey literature. In two project meetings with practitioners and experts in palliative care the development process of a QI set was finalised and the QIs were categorized in a framework, covering the recommendations of the Council of Europe.

**Results:**

The searches resulted in 151 structure and process indicators, which were discussed in steering group meetings. Of those QIs, 110 were eligible for the final framework.

**Conclusions:**

We developed the first set of QIs for the organisation of palliative care. This article is the first step in a multi step project to identify, validate and pilot QIs.

## Background

Following the 2002 definition of the World Health Organisation (WHO), palliative care is no longer restricted to patients with cancer; it should be available for all patients with life-threatening diseases [1]. Furthermore, palliative care is applicable early in the course of the disease and can be delivered in conjunction with interventions that aim to prolong life. Palliative care needs a team approach in order to relieve not only pain and other somatic symptoms but also to provide multi-dimensional care including psychosocial and spiritual care and support for patients and their proxies. This wider definition implies an increase of the number of patients eligible for palliative care. Due to successful medical interventions, the aging population and improved survival of patients with chronic diseases or with cancer, the demand for palliative care will increase too [2,3].

In 2003, the Council of Europe launched recommendations for the organisation of palliative care regarding settings and services, policy and organisation, quality improvement and research, education and training, family, communication with the patient and family, teams and bereavement. This included further cooperation between European countries [4]. As most scientific studies focus on clinical outcomes, it is unclear whether these recommendations and the WHO definition have been implemented in the organisation of palliative care in Europe. By measuring the quality of the organisation of palliative care, patients, caregivers and policy makers can monitor whether in their country, specific settings and networks for palliative care meet the recommendations of the council of Europe and of the WHO. This information would give better insight, which is needed for the measurement of the impact of palliative care programs [5].

A valid and reliable method for assessing the quality of the organisation of care is the use of structure and process quality indicators (QIs). QIs are ‘explicitly defined and measurable items referring to the outcomes, processes or structure of care’ [6,7]. In a systematic review published in 2009, clinical indicators appeared to be widely overrepresented over indicators that assess organisational issues of palliative care, and most QIs were developed in and for one specific country or setting [8].

Therefore, we aimed to develop a scientifically sound European set of structure and process QIs, as a first step in quality measurement and improvement.

## Methods

The study, undertaken by partners from seven collaborating countries (Belgium, United Kingdom, France, Germany, Netherlands, Poland and Spain), ran from October 2007 till September 2010 [9]. It was co-funded by the European Executive Agency for Health and Consumers (EAHC).

QI sets can be based on existing sets of QIs, recommendations from clinical guidelines, scientific literature, best practice or expert consensus [6]. We used a combination of these.

As palliative care, being a relatively young field within health care is changing rapidly. The initial phase of this project was an update and extension of a previous review aiming to find already existing QIs in literature or aspects of the organisation of the palliative care for which QIs would be useful [8]. QIs were operationalized as ‘measurable items referring to the outcomes, processes or structure of care’ [6,7]. Organisation of palliative care was defined as ‘systems to enable the delivery of good quality in palliative care’, which made us focus on processes and structures [7]. Besides publications that describe the development or use of QIs for the organisation of palliative care, publications were used that describe the structure or process of good palliative care, in order to develop QIs if not available yet.

### Main database search

As an update and extension of an existing systematic review, the following bibliographic databases were searched: Medline, Scopus, PsycINFO, Social Medicine, CINAHL, the Cochrane Database, Embase, SIGLE, ASCO, and Google Scholar by an existing search strategy (Additional file 1: Appendix A) [8]. If applicable, Mesh terms were changed, as these are database-specific.

Inclusion criteria were a publication period from December 2007 to May 2009, as the systematic review ran until December 2007 and containing information about the development or use of (sets of) QIs.

Papers describing QIs about palliative care for children, clinical outcome indicators, patient outcome and on treatment were excluded, as well as scientific papers that were not written in English.

The initial selection process was based on independent screening by three researchers of title and/or abstract, followed by a selection based on full text. Additionally, reference lists of obtained papers were studied and hand searches were performed (Current Opinion in Supportive and Palliative Care, Journal of Pain and Symptom Management, Palliative Medicine and Quality and Safety in Health Care Journal).

The QIs derived from the search were categorized in a framework. It was based on (1) a previously developed framework for evalution of the organisation of general practice and adapted for palliative care and (2) the recommendations of the Council of Europe [4,10]. It contains the domains 1. Definition of a palliative care service, 2. Access to palliative care, 3. Infrastructure, 4. Assessment tools, 5. Personnel, 6. Documentation of clinical data, 7. Quality and safety issues, 8. Reporting clinical activity of palliative care, 9. Research and 10. Eduation.

### Grey literature search

If a domain or subdomain of the framework was not covered with QIs found in the literature search, an additional grey literature search was performed. Grey literature was defined as ‘literature which has not been formally published in peer- reviewed literature’ [11]. Inclusion of grey literature was restricted to reports from government agencies or scientific research groups, white papers and websites from national organisations of the seven participating countries. Finally, the network of the Europall research group was used to identify relevant papers.

### Methods of screening and article selection

The steering group of the Europall project planned two meetings in September and October 2009 with all project members (Additional file 1: Appendix B).

### QI selection

The draft set of structure and process QIs was discussed during the first steering group meeting in September 2009. Academic experts from several disciplines in palliative care, all from one of the seven participating European countries were invited. Consensus was based on 1. whether it considered a process or structure QI 2. whether it overlapped with other proposed QIs, 3. to which domain of the framework (Table 1) it belonged [10] and 4. for which settings it was applicable. Based on the grey literature search, the project partners could suggest new QIs about aspects that were relevant but not yet operationalised as QIs.

**Table 1:** Quality indicator set

**Definition of a palliative care service**				
1	All the services below are part of a comprehensive palliative care service: Palliative day care, Palliative home care support team, Hospice beds, Palliative hospital support team, Inpatient palliative care hospital beds, Palliative care outpatient clinic, Bereavement support	Structure indicator	All settings	New developed
2	All the services below are part of a comprehensive palliative care service: Palliative day care	Structure indicator	All settings	New developed
3	All the services below are part of a comprehensive palliative care service: Palliative home care support team	Structure indicator	All settings	New developed
4	All the services below are part of a comprehensive palliative care service: Hospice beds	Structure indicator	All settings	New developed
5	All the services below are part of a comprehensive palliative care service: Palliative hospital support team	Structure indicator	All settings	New developed
6	All the services below are part of a comprehensive palliative care service: Inpatient palliative care hospital beds (e.g. palliative care unit)	Structure indicator	All settings	New developed
7	All the services below are part of a comprehensive palliative care service: Palliative care outpatient clinic	Structure indicator	All settings	New developed
8	All the services below are part of a comprehensive palliative care service: Bereavement support	Structure indicator	All settings	New developed
Access to palliative care				
A. Access and availability (All settings)				
9	A palliative care team is available at the request of the treating professional/team in all of the following settings: Day care, at home, Hospital, Hospice, Nursing home, Outpatient clinic, Day care	Process indicator	All settings	New developed
10	A palliative care team is available at the request of the treating professional/team in all of the following settings: Day care (excluding palliative day care)	Process indicator	All settings	New developed
11	A palliative care team is available at the request of the treating professional/team in all of the following settings: At home (or home replacing institution s.a mental institution, prison)	Process indicator	All settings	New developed
12	A palliative care team is available at the request of the treating professional/team in all of the following settings: Hospital	Process indicator	All settings	New developed
13	A palliative care team is available at the request of the treating professional/team in all of the following settings: Hospice	Process indicator	All settings	New developed
14	A palliative care team is available at the request of the treating professional/team in all of the following settings: Care home	Process indicator	All settings	New developed
15	A palliative care team is available at the request of the treating professional/team in all of the following settings: Outpatient clinic (excluding palliative care outpatient clinic)	Process indicator	All settings	New developed
16	For every professional/team specialised palliative care advice is available 24 hours a day, 7 days a week	Process indicator	All settings	Changed
17	Patients in need of palliative care and their families have access to palliative care facilities: Throughout the entire duration of their disease	Process indicator	All settings	Changed
18	Patients in need of palliative care and their families have access to palliative care facilities: With no extra financial consequences for the patient	Process indicator	All settings	Changed
19	Patients receiving palliative care have access to diagnostic investigations (e.g. X-rays, blood samples) regardless of their setting	Process indicator	All settings	Changed
Primary care (Home, Nursing home)				
20	Palliative care is available for the patient and their family by:Phone	Process indicator	Primary care indicator	Changed
21	Palliative care is available for the patient and their family by: Visiting the patient	Process indicator	Primary care indicator	Changed
22	Palliative care is available for the patient and their family by: Bringing the patient to the service	Process indicator	Primary care indicator	Changed
23	For a palliative patient in a crisis, the following can be arranged within 24 hours: Admission	Process indicator	Primary care indicator	Changed
24	For a palliative patient in a crisis, the following can be arranged within 24 hours: An urgent discharge to patients home	Process indicator	Primary care indicator	Changed
25	For a palliative patient in a crisis, the following can be arranged within 24 hours: Transfer to another setting of care	Process indicator	Primary care indicator	Changed
B. Out of hours (All settings)				
Staff				
26	A member of a palliative care team is available 24 hours a day, 7 days a week: For palliative care consultation by phone	Process indicator	All settings	Changed
27	A member of a palliative care team is available 24 hours a day, 7 days a week: To provide bedside care in a crisis	Process indicator	All settings	Changed
Drugs				
28	The following treatments are available for a palliative patient 24 hours a day, 7 days a week: Opioids and other controlled drugs	Structure indicator	Primary care indicator	Combined/ Changed
29	The following treatments are available for a palliative patient 24 hours a day, 7 days a week: Anticipatory medication for the dying patient	Structure indicator	Primary care indicator	Combined/ Changed
30	The following treatments are available for a palliative patient 24 hours a day, 7 days a week: Syringe drivers	Structure indicator	Primary care indicator	Combined/ Changed
C. Continuity of care (All settings)				
31	There is a procedure for exchange of clinical information across caregivers, disciplines and settings	Process indicator	All settings	Changed
32	Before discharge/transfer/admission there is information transfer to the caregivers in the next setting regarding care and treatment	Process indicator	All settings	Changed
33	There is a professional caregiver per individual palliative patient nominated as responsible ‘key worker‘ who coordinates care	Process indicator	All settings	Combined/ Changed
34	The responsible ‘key worker‘ pays special attention to continuity of care within and across settings	Process indicator	All settings	Combined/ Changed
Inpatient setting (Hospital, Palliative care unit, Hospice)				
35	General practitioners (GP‘s) are routinely called when a patient is being discharged home or transferred to another setting	Process indicator	Inpatient setting indicator	Changed
36	The discharge/transfer letter of palliative care patients contains a multidimensional diagnosis, prognosis and treatment plan (see indicator 48 Clinical record )	Structure indicator	Inpatient setting indicator	Changed
Primary care				
37	The primary care out-of-hours service has handover forms (written or -electronic) with clinical information of all palliative care patients in the terminal phase at home	Structure indicator	Primary care indicator	Changed
Infrastructure				
A. All settings				
Infrastructure				
38	Specialist equipment (e.g. anti decubitus mattresses, aspiration material, stoma care, oxygen delivery, special drug administration pumps, hospital beds, etc.) is available for the nursing care of palliative care patients in each specific setting	Structure indicator	All settings	Changed
39	There is a dedicated room where multidisciplinary team meetings within one setting takes place	Structure indicator	All settings	New developed
40	There are dedicated facilities for multidisciplinary communications across settings: A dedicated room for meetings	Structure indicator	All settings	Changed
41	There are dedicated facilities for multidisciplinary communications across settings: Facilities for video or telephone conferences	Structure indicator	All settings	Changed
Information about care				
42	There is an up to date directory of local caregivers and organisations that can have a role in palliative care	Structure indicator	All settings	New developed
43	There are dedicated information about the palliative care service: A website	Structure indicator	All settings	Changed
44	There are dedicated information about the palliative care service: Leaflets or brochures	Structure indicator	All settings	Changed
45	Patient information should be available in relevant foreign languages	Structure indicator	All settings	Changed
46	Appropriately trained translators should be available if professional caregivers and patient or family members do not speak the same language	Process indicator	All settings	Changed
47	There is a computerised medical record, to which all professional caregivers involved in the care of palliative care patients have access: Within one setting	Process indicator	All settings	Combined
IT systems				
48	There is a computerised medical record, to which all professional caregivers involved in the care of palliative care patients have access: Across different settings	Process indicator	All settings	Combined
B. Inpatient setting (Hospital, Palliative care unit, Hospice, Nursing home)				
49	Consultations with the patient and/or family/informal caregivers are done in an environment where privacy is guaranteed (e.g. there is a dedicated room)	Structure indicator	Inpatient setting indicator	Changed
50	Dying patients are able to have a single bedroom if they want to	Process indicator	Inpatient setting indicator	New developed
51	There are facilities for a relative to stay overnight	Structure indicator	Inpatient setting indicator	New developed
52	Family members and friends are able to visit the dying patient without restrictions of visiting hours	Process indicator	Inpatient setting indicator	Changed
53	There is a private place (e.g. dedicated room) for saying goodbye to the deceased	Structure indicator	Inpatient setting indicator	New developed
C. Home care				
54	For a palliative care patient staying at home there is the possibility, if needed, to provide someone (a volunteer or professional) to stay overnight if needed	Process indicator	Home care indicator	Changed
Assessment tools				
55	There is a holistic assessment of palliative care needs of patients and their family caregivers (e.g. SPARC)	Process indicator	All settings	Changed
56	There is an assessment of pain and other symptoms using a validated instrument	Process indicator	All settings	Changed
Personnel palliative care services				
A. Staff				
57	The multidisciplinary team that provides palliative care consists of at least one of the following disciplines: Physician	Structure indicator	All settings	Changed
58	The multidisciplinary team that provides palliative care consists of at least one of the following disciplines: Nurse	Structure indicator	All settings	Changed
59	The multidisciplinary team that provides palliative care consists of at least one of the following disciplines: Spiritual/religious caregiver	Structure indicator	All settings	Changed
60	The multidisciplinary team that provides palliative care consists of at least one of the following disciplines: Psychologist/Psychiatrist	Structure indicator	All settings	Changed
61	The multidisciplinary team that provides palliative care consists of at least one of the following disciplines: Social worker	Structure indicator	All settings	Changed
62	The multidisciplinary team that provides palliative care consists of at least one of the following disciplines: Physiotherapist	Structure indicator	All settings	Changed
63	The multidisciplinary team that provides palliative care consists of at least one of the following disciplines: Occupational therapist	Structure indicator	All settings	Changed
64	The multidisciplinary team that provides palliative care consists of at least one of the following disciplines: Dietitian	Structure indicator	All settings	Changed
65	The multidisciplinary team that provides palliative care consists of at least one of the following disciplines: Bereavement counselor	Structure indicator	All settings	Changed
66	The multidisciplinary team that provides palliative care consists of at least one of the following disciplines: Pharmacist	Structure indicator	All settings	Changed
B. Education and training for staff/volunteers				
67	New staff receives a standardised induction training	Process indicator	All settings	Changed
68	All team members have certified (accredited?) training in palliative care, appropriate to their discipline	Process indicator	All settings	Changed
69	All volunteers have training in palliative care.	Process indicator	All settings	Combined/ Changed
C. Support systems				
70	All team members have an annual appraisal	Process indicator	All settings	Changed
71	All team members who professionally deal with loss have access to a program for care for the carers	Process indicator	All settings	Changed
72	Satisfaction with working in the team is assessed (e.g. Team Climate Inventory)	Process indicator	All settings	Changed
D. Organisation of care				
73	Palliative care services work in conjunction with the referring professional/team	Process indicator	Inpatient setting indicator	New developed
74	There is a regular interdisciplinary/multi-professional meeting to discuss palliative care patients: daily meetings to discuss day-to- day management of palliative care patients	Process indicator	All settings	Combined/ Changed
75	There is a regular interdisciplinary/multi-professional meeting to discuss palliative care patients:weekly (inter- and multidisciplinary) meeting to review palliative care patients referrals and care plans	Process indicator	All settings	Combined/ Changed
E. Information sharing				
76	All relevant team members are informed about patients who have died	Process indicator	Inpatient setting indicator	Changed
Documentation of clinical data				
A. Clinical record (All settings)				
77	For patients receiving palliative care a structured palliative care clinical record is used	Process indicator	All settings	Changed
78	The palliative care clinical record contains evidence of documentation of the following items: Clinical summary	Process indicator	All settings	Changed
79	The palliative care clinical record contains evidence of documentation of the following items: Physical aspects of care	Process indicator	All settings	Changed
80	The palliative care clinical record contains evidence of documentation of the following items: Psychological and psychiatric aspects of care	Process indicator	All settings	Changed
81	The palliative care clinical record contains evidence of documentation of the following items: Social aspects of care	Process indicator	All settings	Changed
82	The palliative care clinical record contains evidence of documentation of the following items: Spiritual, religious, existential aspects of care	Process indicator	All settings	Changed
83	The palliative care clinical record contains evidence of documentation of the following items: Cultural aspects of care	Process indicator	All settings	Changed
84	The palliative care clinical record contains evidence of documentation of the following items: Care of imminently dying patient	Process indicator	All settings	Changed
85	The palliative care clinical record contains evidence of documentation of the following items: Ethical, legal aspects of care	Process indicator	All settings	Changed
86	The palliative care clinical record contains evidence of documentation of the following items: Multidimensional treatment plan	Process indicator	All settings	Changed
87	The palliative care clinical record contains evidence of documentation of the following items: Follow up assessment	Process indicator	All settings	Changed
B. Timely documentation				
Inpatient setting (Hospital, Palliative care unit, Hospice, Nursing home)				
88	Within 24 hours of admission there is documentation of the initial assessment of: Prognosis, Functional status, Pain and other symptoms, Psychosocial symptoms, The patient‘s capacity to make decisions	Process indicator	Inpatient setting indicator	Changed
89	There is documentation that patients reporting pain or other symptoms at the time of admission, had their pain or other symptoms relieved or reduced to a level of their satisfaction within 48 hours of admission	Process indicator	Inpatient setting indicator	Changed
90	There is documentation about the discussion of patient preferences within 48 hours of admission	Process indicator	Inpatient setting indicator	Changed
91	A discharge/transfer summary is available in the medical record within 48 hours after discharge/transfer	Process indicator	Inpatient setting indicator	Changed
All settings				
92	There is documentation of pain assessment at 4 hour intervals	Process indicator	All settings	Changed
93	The discussion of patient‘s preferences is reviewed on a regular basis (in parallel with disease progression) or on request of the patient	Process indicator	All settings	Changed
94	There is documentation that within 24 hours after patient transfer, the responsible physician in the receiving setting has visited the patient	Process indicator	All settings	Changed
95	There is documentation that within 24 hours after patient transfer, the new palliative care team in the receiving setting has visited the patient	Process indicator	All settings	Changed
Quality and safety issues				
A. Quality policies				
96	The palliative care service has a quality improvement program	Process indicator	All settings	Changed
97	There is documentation whether targets set for quality improvement have been met	Process indicator	All settings	Changed
98	Clinical audit are part of the quality improvement program	Process indicator	All settings	Changed
99	The setting uses a program about early initiation of palliative care (e.g. the Gold Standards Framework)	Process indicator	All settings	Changed
B. Adverse events				
100	There is a register for adverse events	Process indicator	All settings	Changed
101	There is a documented procedure to analyse and follow up adverse events	Process indicator	All settings	Changed
C. Complaints procedure				
102	There is a patient complaints procedure	Process indicator	All settings	Changed
Reporting clinical activity of palliative care services				
103	The palliative care service uses a database for recording clinical activity	Process indicator	All settings	Changed
104	The following is part of the database: Diagnosis, Date of diagnosis, Date of referral, Date of admission to the palliative care service, Date of death, Place of death, Preferred place of death	Process indicator	All settings	Changed
105	From the database the service is able to derive: Time from diagnosis to referral to palliative care, Time from referral to initiation of palliative care, Time from initiation of palliative care to death, Frequency of unplanned consultations with the out-of-hours service for palliative care patients who are at home, Frequency of unplanned hospital admissions of palliative care patients, Percentage of non-oncological patients receiving palliative care	Process indicator	All settings	New developed
106	Based on the database, an annual report is made about the service	Process indicator	All settings	Changed
Research				
107	There is evidence that the palliative care service is involved in research in palliative care (e.g. authorship of publications, research grants)	Process indicator	All settings	Changed
Education				
108	All health and social care students have standardised learning objectives for basic training in palliative care	Process indicator	All settings	Changed
109	All health and social care professionals have standardised learning objectives for continuing basic training in palliative care	Process indicator	All settings	New developed
110	There is a program for specialised training in palliative care for professionals working in a service that provides specialised palliative care	Process indicator	All settings	New developed

3. Based on this meeting, adaptations were made and a new draft QI set was presented in the second steering group meeting in October.

## Results

### Search flow

The literature search resulted in 541 papers, including a previous systematic review on quality indicators for palliative care [8]. Most of the papers came from the database search (n=527), followed by the hand search (n= 29) and least of grey literature search (n=14).

**Figure 1:**
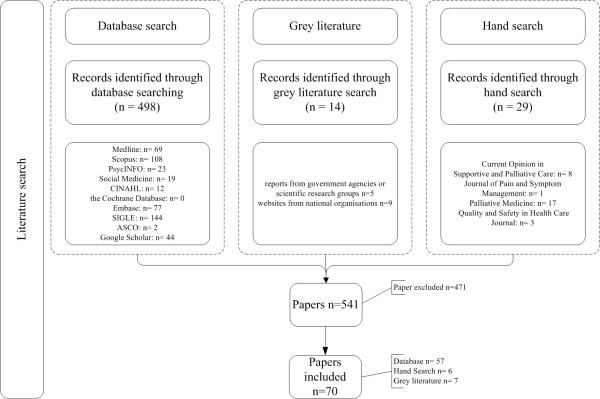
Flow chart literature search.

In the screening process 16 duplicates were identified, and titles and abstracts of 511 papers were searched. Of these, 389 documents were excluded, as they did not contain QIs. Full papers were obtained of 122 publications, from which 63 papers were included; 57 resulting from the database search [12-68] and another six papers from the additional hand searches (Figure 1) [69-74]. 

### Results grey literature search

The grey literature search yielded seven papers, deriving from Belgium, the Netherlands and the UK [9,75-80]. These sources included government sites, national health organisations and national institutes (Figure 1). This additional search resulted in the development of 53 QIs, divided over almost all domains (see Additional file 1).

### QI development

**Figure 2:**
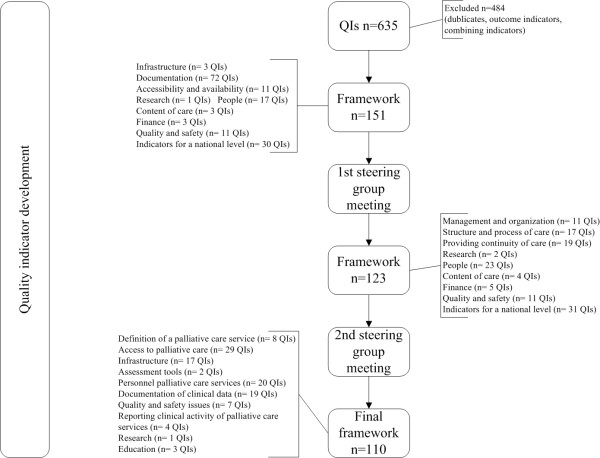
Flow chart quality indicator development.

Sixhundred-thirtyfive QIs were derived from this literature review. After screening of duplicates, selecting process and structure QIs and combining QIs covering the same topic, the remaining 151 QIs were organised in the framework and discussed in the first steering group meeting. The two steering group meetings resulted in a reduction from 151 to 110 QIs (Additional file 1: Appendix C) (Figure 2). For instance the domain about finance QIs was excluded for the final set as the QIs were more useful on national level than in the setting specific palliative care institutions.

The rest of the QIs were distributed over the framework (Table 1) [10].

The majority of the 110 QIs were process QIs (n=76), the other structure QIs (n=34). Some of the QIs (n=24) were only applicable in specific settings; ten in primary care, thirteen in inpatient settings and one in home care. The others were meant for all settings that deliver palliative care.

Twenty-four QIs were developed based on organisational aspects found in literature (Table 1, QI 51). Finally, several QIs (n= 86), were changed in their presentation of text during the procedure. For example, originally developed QIs for other settings like the intensive care unit, were adapted to make them appropriate for palliative care settings.

## Discussion

We were able to develop an international framework with 110 QIs to assess the organisation of palliative care in several kind of settings. To our knowledge, this study presents the first systematically developed international set of QIs on this topic. Part of the QIs are setting specific, whereas others will be applicable in all kind of settings that deliver palliative care.

Where Pasman et al. performed a systematic review on all kind of QIs for palliative care, and Pastrana et al. focused on outcome indicators for Germany, we focused on process and structure QIs [8,81]. By using an international perspective and by not limiting the study to symptom control, our study follows the recommendations of Ostgathe et al. [82]. Our set also contains two QIs that are linked to the World Health Assembly’s proposed global health indicator ‘Access to palliative care assessed by morphine-equivalent consumption of strong opioid analgesia (excluding methadone) per death by cancer’, but without the restriction to patients with cancer [83].

### Strength and limitations

We chose an approach with several consecutive methodological steps to develop a set of QIs. Of those aspects that were considered important for the organisation of palliative care but of which no QIs could be found, we developed QIs ourselves [84]. Of those QIs that were developed for a restricted group of patients or setting (e.g. ICU or vulnerable elderly) we checked whether we could rephrase them into QIs for more types of settings or palliative patients. Defining QIs in a consensus procedure is a good option if scientific literature is not yet available [7], particularly because it combines several methods to improve validity. Using a group approach has the advantage that participants can share their expertise and experience. Groups often make better decisions than individuals [85].

The naming of QIs as process or structure indicators can be discussed. Yet, this only influences the categorisation and not the content, importance or use of a QI.

Another strong aspect of our procedure is the inclusion of grey literature, which created the possibility to include documents from important although not scientific sources [86].

As the Europall project was a collaboration of seven European countries, only experts of these countries were represented in the steering group meetings. Other European countries, with different health care and financing systems, cultures and palliative care, were not involved at this stage.

This first step resulted in a set of structure and process QIs, that can help professionals or settings to measure the quality of care of their setting. In a next step, a subset will be developed of which each QI is applicable in the seven participating countries.

Based on a modified RAND Delphi method the following set will be interesting for international comparison. The advantage of this comprehensive set enables each country and each setting the opportunity to see all QIs that are available on this topic.

The last step will describe a pilot study to test the set of QIs on face-validity, applicability and discriminative power. This includes almost all (26) European countries. These studies will be published separately.

### Further research

The final set can be used to provide feedback to settings or countries to reflect on their performance, for supporting quality improvement activities, accreditation, research, and enhancing transparency about quality. They can be used to evaluate the implementation of the WHO definition and the recommendations of the council of Europe [1,4].

From 2011 to 2015, a follow-up project to Europall called IMPACT (funded by the EU 7^th^ framework) will develop and test strategies to implement these QIs.

## Conclusions

This review resulted in the first comprehensive framework of QIs for the organisation of palliative care.

## Competing interest

This work was partly funded by EAHC (Executive Agency for Health and Consumers, grant: 2006111 PPP ‘Best practices in palliative care’). The funders had no role in study design, data collection and analysis, decision to publish, or preparation of the manuscript. The authors have no financial disclosures.

## Authors' contributions

KvB participated in the literature search, design of the study and drafted the manuscript. NA participated in the literature search, design of the study and drafted the manuscript. JH participated in the literature search, design of the study and drafted the manuscript. JMM was actively involved in the selection and developmental process of the QI. She attended the expert meeting. IC was actively involved in the selection and developmental process of the QI. She attended the expert meeting. LR and helped to draft the manuscript and had an advisory role. KV conceived of the study and participated in its design and coordination and helped to draft the manuscript. YE conceived of the study and participated in its design and coordination and helped to draft the manuscript. All authors read and approved the final manuscript.

## Pre-publication history

The pre-publication history for this paper can be accessed here:

http://www.biomedcentral.com/1472-6963/12/381/prepub

## Supplementary Material

Additional file 1 **Supplementary online content.** Development of a set of process and structure indicators for palliative care: the Europall project. Appendix A- Search strategies for databases. Appendix B- Project partners. Appendix C- Indicators set for the organisation of palliative care. Click here for file

## References

[B1] WHO Definition of Palliative Care http://www.who.int/cancer/palliative/definition/en/

[B2] 10 facts on ageing and the life course http://www.who.int/features/factfiles/ageing/en/index.html

[B3] What are the public health implications of global ageing? http://www.who.int/features/qa/42/en/index.html

[B4] Recommendation Rec (2003) 24 of the Committee of Ministers to member states on the organisation of palliative care http://www.coe.int/t/dg3/health/Source/Rec(2003)24_en.pdf

[B5] Smith TJ Hillner BE Ensuring quality cancer care by the use of clinical practice guidelines and critical pathways J Clin Oncol 2001 19 2886 2897 11387362 10.1200/JCO.2001.19.11.2886

[B6] Campbell SM Ludt S Van Lieshout J Boffin N Wensing M Petek D Grol R Roland MO Quality indicators for the prevention and management of cardiovascular disease in primary care in nine European countries Eur J Cardiovasc Prev Rehabil 2008 15 509 515 10.1097/HJR.0b013e328302f44d 18695594

[B7] Campbell SM Braspenning J Hutchinson A Marshall MN Research methods used in developing and applying quality indicators in primary care BMJ 2003 326 816 819 10.1136/bmj.326.7393.816 12689983 PMC1125721

[B8] Pasman HRW Brandt HE Deliens L Francke AL Quality indicators for palliative care: a systematic review J Pain Symptom Manage 2009 38 145 156 10.1016/j.jpainsymman.2008.07.008 19615636

[B9] Europall Project http://www.europall.eu/

[B10] Engels Y Campbell S Dautzenberg M van den Hombergh P Brinkmann H Szecsenyi J Falcoff H Seuntjens L Kuenzi B Grol R Developing a framework of, and quality indicators for, general practice management in Europe Fam Pract 2005 22 215 222 10.1093/fampra/cmi002 15722398

[B11] Cook AM Finlay IG Edwards AG Hood K Higginson IJ Goodwin DM Normand CE Douglas HR Efficiency of searching the grey literature in palliative care Journal of Pain and Symptom Management 2001 22 797 801 10.1016/s0885-3924(01)00315-3 11532593

[B12] Aa Petersen M Pedersen L Groenvold M Does the agreement of patient and physician assessments of health related quality of life in palliative care depend on patient characteristics? Palliat Med 2007 21 289 294 10.1177/0269216307077694 17656405

[B13] ASCO-ESMO Consensus statement on quality cancer care J Clin Oncol 2010 24 3498 3499 10.1200/JCO.2006.07.4021 16751437

[B14] Becker G Sarhatlic R Olschewski M Xander C Momm F Blum HE End-of-life care in hospital: current practice and potentials for improvement J Pain Symptom Manage 2007 33 711 719 10.1016/j.jpainsymman.2006.09.030 17531911

[B15] Bottomley A The journey of health-related quality of life assessment Lancet Oncol 2008 9 906 10.1016/S1470-2045(08)70233-X 18760247

[B16] Brumley R Enguidanos S Jamison P Seitz R Morgenstern N Saito S McIlwane J Hillary K Gonzalez J Increased satisfaction with care and lower costs: results of a randomized trial of in-home palliative care J Am Geriatr Soc 2007 55 993 1000 10.1111/j.1532-5415.2007.01234.x 17608870

[B17] Casarett D Pickard A Bailey FA Ritchie C Furman C Rosenfeld K Shreve S Chen Z Shea JA Do palliative consultations improve patient outcomes? J Am Geriatr Soc 2008 56 593 599 10.1111/j.1532-5415.2007.01610.x 18205757

[B18] Delgado-Guay MO Parsons HA Li Z Palmer LJ Bruera E Symptom distress, interventions, and outcomes of intensive care unit cancer patients referred to a palliative care consult team Cancer 2009 115 437 445 10.1002/cncr.24017 19107768

[B19] Demiris G Oliver DP Wittenberg-Lyles E Assessing caregivers for team interventions (ACT): a new paradigm for comprehensive hospice quality care Am J Hosp Palliat Care 2009 26 128 134 10.1177/1049909108328697 19116302 PMC2666779

[B20] Dixon BK Quality measures crafted for palliative. Hospice Medicine Family Practice News 2007 37 6

[B21] Dougherty E Pierce B Ma C Panzarella T Rodin G Zimmermann C Factors associated with work stress and professional satisfaction in oncology staff Am J Hosp Palliat Care 2009 26 105 111 10.1177/1049909108330027 19144993

[B22] Dy SM Shugarman LR Lorenz KA Mularski RA Lynn J for the RAND—Southern California Evidence-Based Practice Center A systematic review of satisfaction with care at the end of life J Am Geriatr Soc 2008 56 124 129 10.1111/j.1532-5415.2007.01507.x 18031485

[B23] Gelfman LP Meier DE Morrison RS Does palliative care improve quality? A survey of bereaved family members J Pain Symptom Manage 2008 36 22 28 10.1016/j.jpainsymman.2007.09.008 18411019 PMC2527760

[B24] Gomez-Batiste X Fontanals MD Roca J Borras JM Viladiu P Stjernsward J Rius E Catalonia WHO demonstration project on palliative care implementation 1990–1995: results in 1995 J Pain Symptom Manage 1996 12 73 78 10.1016/0885-3924(96)00074-7 8754983

[B25] Granda-Cameron C Viola SR Lynch MP Polomano RC Measuring patient-oriented outcomes in palliative care: functionality and quality of life Clin J Oncol Nurs 2008 12 65 77 10.1188/08.CJON.65-77 18258576

[B26] Gries CJ Curtis JR Wall RJ Engelberg RA Family member satisfaction with end-of-life decision making in the ICU Chest 2008 133 704 712 10.1378/chest.07-1773 18198256 PMC2744978

[B27] Griffin JP Koch KA Nelson JE Cooley ME Palliative care consultation, quality-of-life measurements, and bereavement for end-of-life care in patients with lung cancer: ACCP evidence-based clinical practice guidelines (2nd edition) Chest 2007 132 404S 422S 10.1378/chest.07-1392 17873182

[B28] Grunfeld E Lethbridge L Dewar R Lawson B Paszat LF Johnston G Burge F McIntyre P Earle CC Towards using administrative databases to measure population-based indicators of quality of end-of-life care: testing the methodology Palliat Med 2006 20 769 777 10.1177/0269216306072553 17148531 PMC3741158

[B29] Hanratty B Holland P Jacoby A Whitehead M Financial stress and strain associated with terminal cancer–a review of the evidence Palliat Med 2007 21 595 607 10.1177/0269216307082476 17942498

[B30] Hicks F Rees E A ‘pain-free‘ death Br Med Bull 2008 88 23 41 10.1093/bmb/ldn043 19036805

[B31] Irwin SA Zurhellen CH Diamond LC Dunn LB Palmer BW Jeste DV Twamley EW Unrecognised cognitive impairment in hospice patients: a pilot study Palliat Med 2008 22 842 847 10.1177/0269216308096907 18772210 PMC4047032

[B32] Jarabek BR Jama AA Cha SS Ruegg SR Moynihan TJ McDonald FS Use of a palliative care order set to improve resident comfort with symptom management in palliative care Palliat Med 2008 22 343 349 10.1177/0269216308090169 18541638

[B33] Jordhoy MS Inger Ringdal G Helbostad JL Oldervoll L Loge JH Kaasa S Assessing physical functioning: a systematic review of quality of life measures developed for use in palliative care Palliat Med 2007 21 673 682 10.1177/0269216307083386 18073253

[B34] Jünger S Pestinger M Elsner F Krumm N Radbruch L Criteria for successful multiprofessional cooperation in palliative care teams Palliat Med 2007 21 347 354 10.1177/0269216307078505 17656412

[B35] Kairuz TE Gargiulo D Bunt C Garg S Quality, safety and efficacy in the ‘off-label‘ use of medicines Curr Drug Saf 2007 2 89 95 10.2174/157488607779315471 18690954

[B36] Kapo J Morrison LJ Liao S Palliative care for the older adult J Palliat Med 2007 10 185 209 10.1089/jpm.2006.9989 17298269

[B37] Kim Y Given BA Quality of life of family caregivers of cancer survivors: across the trajectory of the illness Cancer 2008 112 2556 2568 10.1002/cncr.23449 18428199

[B38] Le BHC Ashby MA Audit of deaths and palliative care referrals in a large Australian teaching hospital J Palliat Med 2007 10 835 836 10.1089/jpm.2007.0034 17803397

[B39] Leonard M Agar M Mason C Lawlor P Delirium issues in palliative care settings J Psychosom Res 2008 65 289 298 10.1016/j.jpsychores.2008.05.018 18707953

[B40] Lorenz K Progress in measuring and improving palliative and end-of-life quality J Palliat Med 2008 11 682 684 10.1089/jpm.2008.9906 18588397

[B41] Mayadev AS Weiss MD Distad BJ Krivickas LS Carter GT The amyotrophic lateral sclerosis center: a model of multidisciplinary management Phys Med Rehabil Clin N Am 2008 19 619 631 10.1016/j.pmr.2008.04.004 18625420

[B42] McNiff KK Neuss MN Jacobson JO Eisenberg PD Kadlubek P Simone JV Measuring supportive care in medical oncology practice: lessons learned from the quality oncology practice initiative J Clin Oncol 2008 26 3832 3837 10.1200/JCO.2008.16.8674 18688049

[B43] Meraviglia M Sutter R Gaskamp CD Providing spiritual care to terminally ill older adults J Gerontol Nurs 2008 34 8 14 18649819 10.3928/00989134-20080701-08

[B44] Miller SC Kiely DK Teno JM Connor SR Mitchell SL Hospice care for patients with dementia: does volume make a difference? J Pain Symptom Manage 2008 35 283 291 10.1016/j.jpainsymman.2007.12.001 18215499

[B45] Miyashita M Nakamura A Morita T Bito S Identification of quality indicators of end-of-life cancer care from medical chart review using a modified Delphi method in Japan Am J Hosp Palliat Care 2008 25 33 38 10.1177/1049909107307376 18160547

[B46] Norton SA Hogan LA Holloway RG Temkin-Greener H Buckley MJ Quill TE Proactive palliative care in the medical intensive care unit: effects on length of stay for selected high-risk patients Crit Care Med 2007 35 1530 1535 10.1097/01.CCM.0000266533.06543.0C 17452930

[B47] van der Ploeg E Depla MFIA Shekelle P Rigter H Mackenbach JP Developing quality indicators for general practice care for vulnerable elders; transfer from US to The Netherlands Qual Saf Health Care 2008 17 291 295 10.1136/qshc.2007.023226 18678728

[B48] Pronost A Le Gouge ALD The effects of various features of haematology-oncology services using the palliative approach and the socio-demographic characteristics of healthcare providers on health indicators: social support, perceived stress, coping strategies, quality of life at work Oncology 2008 10 125 134 10.1007/s10269-007-0775-1

[B49] Quill TE Is length of stay on hospice a critical quality of care indicator? J Palliat Med 2007 10 290 292 10.1089/jpm.2006.9984 17472496

[B50] Ripamonti CI Malignant bowel obstruction: tailoring treatment to individual patients J Support Oncol 2008 6 114 115 18402301

[B51] Robinson C Pesut B Bottorff J Mowry A Broughton S Fyles G Rural palliative care: a comprehensive review J Palliat Med 2009 12 253 258 10.1089/jpm.2008.0228 19216703

[B52] Rokoske FS Schenck AHL Developing Quality Measures for Hospice and Palliative Care Gerontologist 2008 48 691

[B53] Sampson EL Thune-Boyle I Kukkastenvehmas R Jones L Tookman A King M Blanchard MR Palliative care in advanced dementia; A mixed methods approach for the development of a complex intervention BMC Palliat Care 2008 7 8 10.1186/1472-684X-7-8 18620567 PMC2475530

[B54] Sato K Miyashita M Morita T Sanjo M Shima Y Uchitomi Y Reliability assessment and findings of a newly developed quality measurement instrument: quality indicators of end-of-life cancer care from medical chart review at a Japanese regional cancer center J Palliat Med 2008 11 729 737 10.1089/jpm.2007.0227 18588405

[B55] Sela RA Screening for depression in palliative cancer patients attending a pain and symptom control clinic Palliat Support Care 2007 5 207 217 17969824 10.1017/s1478951507000375

[B56] Slaven M Wylie N Fitzgerald B Henderson N Taylor S Who needs a palliative care consult?: the Hamilton Chart Audit tool J Palliat Med 2007 10 304 307 10.1089/jpm.2006.0237 17472499

[B57] Smith AK McCarthy EP Paulk E Balboni TA Maciejewski PK Block SD Prigerson HG Racial and ethnic differences in advance care planning among patients with cancer: impact of terminal illness acknowledgment, religiousness, and treatment preferences J Clin Oncol 2008 26 4131 4137 10.1200/JCO.2007.14.8452 18757326 PMC2654372

[B58] van der Steen JT Mitchell SL Frijters DHM Kruse RL Ribbe MW Prediction of 6-month mortality in nursing home residents with advanced dementia: validity of a risk score J Am Med Dir Assoc 2007 8 464 468 10.1016/j.jamda.2007.05.004 17845950

[B59] Tei Y Morita T Nakaho T Takigawa C Higuchi A Suga A Tajima T Ikenaga M Higuchi H Shimoyama N Fujimoto M Treatment efficacy of neural blockade in specialized palliative care services in Japan: a multicenter audit survey J Pain Symptom Manage 2008 36 461 467 10.1016/j.jpainsymman.2007.11.009 18504097

[B60] Teno JM Connor SR Referring a patient and family to high-quality palliative care at the close of “life: “We met a new personality… with this level of compassion and empathy“JAMA 2009 301 651 659 10.1001/jama.2009.109 19211472

[B61] Teunissen SCCM Verhagen EH Brink M van der Linden BA Voest EE de Graeff A Telephone consultation in palliative care for cancer patients: 5 years of experience in The Netherlands Support Care Cancer 2007 15 577 582 10.1007/s00520-006-0202-y 17165090

[B62] Torres-Vigil I Aday LA Health care providers‘ assessments of the quality of advanced-cancer care in Latin American medical institutions: a comparison of predictors in five countries: Argentina, Brazil, Cuba, Mexico, and Peru J Pain Palliat Care Pharmacother 2008 22 7 20 10.1080/15360280801989195 19042817

[B63] Tsai LY Li IF Liu CP Su WH Change TY Application of quality audit tools to evaluate care quality received by terminal cancer patients admitted to a palliative care unit Support Care Cancer 2008 16 1067 1074 10.1007/s00520-007-0365-1 18196292

[B64] Tuffrey-Wijn I Hogg JCL End-of-life and palliative care for people with intellectual disabilities who have cancer or other life-limiting illness: a review of the literature and available resources J Appl Res Intellect Disabil 2007 20 331 344 10.1111/j.1468-3148.2006.00350.x

[B65] Walling A Lorenz KA Dy SM Naeim A Sanati H Asch SM Wenger NS Evidence-based recommendations for information and care planning in cancer care J Clin Oncol 2008 26 3896 3902 10.1200/JCO.2007.15.9509 18688058

[B66] Wenig LC Huang HL Wilkie DJ Hoenig NA Suarez ML Marschke M Durham J Predicting survival with the palliative performance scale in a minority-serving hospice and palliative care program J Pain Symptom Manage 2009 37 642 648 10.1016/j.jpainsymman.2008.03.023 18823751 PMC2699378

[B67] Yao CA Hu WY Lai YF Cheng SY Chen CY Chiu TY Does dying at home influence the good death of terminal cancer patients? J Pain Symptom Manage 2007 34 497 504 10.1016/j.jpainsymman.2007.01.004 17629664

[B68] Zib M Saul P A pilot audit of the process of end-of-life decision-making in the intensive care unit Crit Care Resusc 2007 9 213 218 17536994

[B69] Costantini M Editorial: place of death. It is time for a change of gear Palliat Med 2008 22 785 786 10.1177/0269216308096424 18838490

[B70] Dudgeon DJ Knott C Eichholz M Gerlach JL Chapman C Viola R Van Dijk J Preston S Batchelor D Bartfay E Palliative Care Integration Project (PCIP) quality improvement strategy evaluation J Pain Symptom Manage 2008 35 573 582 10.1016/j.jpainsymman.2007.07.013 18358693 PMC7125855

[B71] Glasgow JL McLennan SR High KJ Celi LAG Quality of dying in a New Zealand teaching hospital Qual Saf Health Care 2008 17 244 248 10.1136/qshc.2007.024745 18678719

[B72] Luthy C Cedraschi C Pautex S Rentsch D Piguet V Allaz AF Difficulties of residents in training in end-of-life care. A qualitative study Palliat Med 2009 23 59 65 18996979 10.1177/0269216308098796

[B73] Rayner L Loge JH Wasteson E Higginson IJ The detection of depression in palliative care Curr Opin Support Palliat Care 2009 3 55 60 10.1097/SPC.0b013e328326b59b 19365162

[B74] Zaider T Kissane D The assessment and management of family distress during palliative care Curr Opin Support Palliat Care 2009 3 67 71 10.1097/SPC.0b013e328325a5ab 19365164 PMC5557503

[B75] Externe indicatoren voor pijn bij kanker http://www.zichtbarezorg.nl/mailings/FILES/htmlcontent/Ziekenhuizen/New%202e%20tranche/Pijn%20bij%20kanker%20defnew%20incl%20wijzigingen%20nav%20autorisatie.pdf

[B76] Klinische kwaliteitsindicatoren. Objective Elements - Communication (OEC). Brussel: Federaal Kenniscentrum voor de gezondheidszorg (KCE) https://kce.fgov.be/sites/default/files/page_documents/d20061027343.pdf

[B77] Improving Supportive and Palliative Care for Adults National Institute for Clinical Excellence with Cancer http://www.nice.org.uk/nicemedia/live/10893/28816/28816.pdf

[B78] Plan van Aanpak Palliatieve Zorg 2008–2010 http://www.palliatief.nl/LinkClick.aspx?fileticket=yX68iRdBUjw%3d&tabid=3997&mid=10542

[B79] Quality and outcomes framework http://www.paymodernisation.scot.nhs.uk/gms/quality/docs/QualOutFrame0804.pdf

[B80] Studie inzake de ontwikkeling van een registratie-instrument voor palliatieve zorg http://www.palliatief.be/accounts/143/attachments/Research/studie_registratie-instrument_pz_def.versie.pdf

[B81] Pastrana T Radbruch L Nauck F Hover G Fegg M Pestinger M Ross J Krumm N Ostgathe C Outcome indicators in palliative care–how to assess quality and success. Focus group and nominal group technique in Germany Support Care Cancer 2010 18 859 868 10.1007/s00520-009-0721-4 19701782 PMC3128732

[B82] Ostgathe C Voltz R Quality indicators in end-of-life care Curr Opin Support Palliat Care 2010 4 170 173 10.1097/SPC.0b013e32833add10 20489644

[B83] Payne S Leget C Peruselli C Radbruch L Quality indicators for palliative care: debates and dilemmas Palliat Med 2012 26 679 680 10.1177/0269216312450123 22733962

[B84] Claessen S Francke A Belarbi H Pasman H van der Putten M Deliens L A new set of quality indicators for palliative care: process and results of the development trajectory J Pain Symptom Manage 2011 42 169 182 10.1016/j.jpainsymman.2010.10.267 21429703

[B85] Mrowietz U Kragballe K Reich K Spuls P Griffiths C Nast A Franke J Antoniou C Arenberger P Balieva F Bylaite M Correia O Dauden E Gisondi P Iversen L Kemeny L Lahfa M Nijsten T Rantanen T Reich A Rosenbach T Segaert S Smith C Talme T Volc-Platzer B Yawalkar N Definition of treatment goals for moderate to severe psoriasis: a European consensus Arch Dermatol Res 2010 303 1 10 20857129 10.1007/s00403-010-1080-1 PMC3016217

[B86] Genet N Boerma WG Kringos DS Bouman A Francke AL Fagerstrom C Melchiorre MG Greco C Deville W Home care in Europe: a systematic literature review BMC Health Service Research 2011 11 207 10.1186/1472-6963-11-207 PMC3170599 21878111

