# Supplementary material for: Development of a set of process and structure indicators for palliative care: the Europall project
Source: BMC Health Serv Res. 2012 Nov 2;12:381. doi: 10.1186/1472-6963-12-381 (PMC3529116; doi:10.1186/1472-6963-12-381)
Supplement: Additional file 1 — Supplementary online content. Development of a set of process and structure indicators for palliative care: the Europall project. Appendix A- Search strategies for databases. Appendix B- Project partners. Appendix C- Indicators set for the organisation of palliative care. [file 1472-6963-12-381-S1.doc]

Supplementary online content:

Title: ***Development of a set of process and structure indicators for palliative care: the Europall project***

Manuscript ID:  2050296145621786

Overview Page

Appendix A- Search strategies for databases 2

Appendix B- Project partners 3

Appendix C- Indicators set for the organisation of palliative care 4

Appendix A- Search strategies for databases

***Table 1 Search strategies for databases***

| **Database** | **Search strategy** |
| --- | --- |
| **Medline** | (((((''Palliative Care''[MeSH] OR ((palliative[ti] OR terminal[ti] OR hospice[ti]) AND care[ti]) OR ''Terminal Care''[MeSH:noexp] OR ''Life Support Care''[MeSH] OR ''Advance Care Planning''[-MeSH] OR ''Resuscitation Orders''[MeSH] OR ''Withholding Treatment''[MeSH] OR ''Hospice Care''[MeSH] OR ''Hospices''[MeSH] OR ''Terminally Ill''[MeSH]))) AND ((''Quality Indicators, Health Care''[MeSH]))) OR ((((''Palliative Care''[MeSH] OR ((palliative[ti] OR terminal[ti] OR hos-pice[ti]) AND care[ti]) OR ''Terminal Care''[MeSH:noexp] OR ''Life Support Care''[MeSH] OR ''Advance Care Planning''[MeSH] OR ''Resuscitation Orders''[MeSH] OR ''Withholding Treatment''[MeSH] OR ''Hospice Care''[MeSH] OR ''Hospices''[MeSH] OR ''Terminally Ill''[-MeSH]))) AND ((''quality measure'' OR ''quality measures'' OR ''quality criterium'' OR ''quality criteria'' OR ''quality assessment'' OR ((Quality[ti] OR performance[ti] OR satisf*[ti]) AND (indicator*[tw] OR criteri*[tw] OR assess*[ti] OR measur*[ti] OR scale[ti] OR validat*[tw])))))) NOT (letter[pt] OR editorial[pt] OR comment[pt] OR case reports[pt]) |
| **Scopus** | See Medline |
| **PsycINFO 18.05.2009** | ((palliative care or terminal care or hospice care or advance care or life support care) and (quality indicators or measure or quality measures or assessment) and (organisation or organization) |
| **Social Medicine 26.05.2009** | "Title=('palliative care' or 'end of life care' or 'hospice care' or hospice or 'terminal care') And Title=('quality measure?' or 'quality assessment' or 'quality criteri?' or indicator?)" in Dates (After 2006) |
| **CINAHL 18.05.2009** | ((“palliative care” OR “terminal care” OR “hospice care” OR “advance care” OR “life support care” OR “end of life care”) AND ("quality indicator*" OR “ clinical indicator*” OR “clinical measure*” OR "quality measure*" OR assessment) AND organi?ation) -"intensive -care -unit" -"symptom -assessment" |
| **Cochrane Database** | See Medline |
| **Embase 15.05.2009** | (exp Palliative Therapy/ OR ((palliative.ti. OR terminal.ti. OR hospice.ti.) AND care.ti.) OR Terminal Care/ OR Life Support Care.mp. OR Advance Care Planning.mp. OR Resuscitation/ OR Witholding Treatment.mp. OR exp Hospice Care/ OR exp Hospice/ OR exp Terminally ill patient/) AND (clinical indicator/ or performance measurement system/ OR quality measure OR quality measures OR quality criterium OR quality criteria OR quality assessment OR ((Quality.ti. OR performance.ti. OR satisf*.ti.) AND (indicator*.mp. OR criteri*.ti. OR assess*.ti. OR measur*.ti. OR scale.ti. OR validat*.mp.))) |
| **SIGLE** | ((palliative care OR terminal care OR hospice care OR advance care OR life support care) AND (quality indicators OR measure OR quality measures OR assessment) AND (organisation))  “palliative care” OR “terminal care” OR “hospice care” OR “advance care” OR “end of life care” and (indicators OR measure OR assessment OR audit) |
| **ASCO** | See Medline |
| **Wonca** | See Medline |
| **Google Scholar** | (“palliative care” OR “terminal care” OR “hospice care” OR “advance care” OR “end of life care” ) AND (indicators OR "quality measure" OR audit) 🡪 5620 results vanaf 2007  (“palliative care” OR “terminal care” OR “hospice care” OR “advance care” OR “end of life care” ) AND (indicators OR "quality measure") 🡪 3 780 results vanaf 2007  (“palliative care” OR “terminal care” OR “hospice care” OR “advance care” OR “end of life care” ) AND (indicators OR "quality measure" OR audit) AND ( organization OR organisation)  ((“palliative care” OR “terminal care” OR “hospice care” OR “advance care” OR “life support care” OR “end of life care”) AND (quality indicators OR measure OR quality measures OR assessment) AND (organization OR organization)) NOT “intensive care unit” 🡪 3810 vanaf 2007  ((“palliative care” OR “terminal care” OR “hospice care” OR “advance care” OR “life support care” OR “end of life care”) AND ("quality indicators" OR indicator OR measure OR "quality measures" OR assessment) AND (organization OR organization)) -"intensive -care -unit" 🡪 4320 vanaf 2007  ((“palliative care” OR “terminal care” OR “hospice care” OR “advance care” OR “life support care” OR “end of life care”) AND ("quality indicator" OR “ clinical indicator” OR “clinical measure” OR "quality measures" OR assessment) AND (organization OR organization)) -"intensive -care -unit" 🡪 3 750 sinds 2007  ((“palliative care” OR “terminal care” OR “hospice care” OR “advance care” OR “life support care” OR “end of life care”) AND ("quality indicator" OR “ clinical indicator” OR “clinical measure” OR "quality measures" OR assessment) AND (organization OR organization)) -"intensive -care -unit" -"symptom -assessment" 🡪3 540 hits  ((“palliative care” OR “terminal care” OR “hospice care” OR “advance care” OR “life support care” OR “end of life care”) AND ("quality indicator" OR “ clinical indicator” OR “clinical measure” OR "quality measures" OR assessment) AND (organization OR organization)) -"intensive -care -unit" – “symptom assessment” |

Appendix B- Project partners

*Belgium:*

Prof. Johan Menten, MD- University Hospital Leuven, Department of Radiotherapy-Oncology and Palliative Medicine, chair in palliative medicine

Karen van Beek- University Hospital Leuven, Department of Radiotherapy-Oncology and Palliative Medicine, radiation-oncologist

*France:*

Jean-Marc Mollard- Réseau Ensemble, Paris, General practitioner

Isabelle Colombet-Sante´Publique Informatique Médicale, INSERM UMRS, eq. 20, Faculté de Médicine de Paris 5, General Practitioner

*Germany:*

Prof. Eberhard Klaschik, Universitätsklinikum der Rheinischen Friedrich-Wilhelms-Universität Bonn, Zentrum für Palliativmedizin, former chair in palliative medicine

Birgit Jaspers- Universitätsklinikum der Rheinischen Friedrich-Wilhelms-Universität Bonn, Zentrum für Palliativmedizin, researcher

*The Netherlands:*

Prof. Kris Vissers, MD- Radboud University Nijmegen Medical Centre, Department of Anaesthesiology, Pain and Palliative Medicine, chair in palliative medicine

Dr Yvonne Engels*-* Radboud University Nijmegen Medical Centre, Department of Anaesthesiology, Pain and Palliative Medicine, assistant professor

Dr Jeroen Hasselar- Radboud University Nijmegen Medical Centre, Department of Anaesthesiology, Pain and Palliative Medicine, assistant professor

Kathrin Woitha- Radboud University Nijmegen Medical Centre, Department of Anaesthesiology, Pain and Palliative Medicine, researcher

*Poland:*

Dr Wojciech Leppert- Poznan University of Medical Science, Department Palliative Medicine, chair in palliative care

Dr Sylwia Dziegielewska, Poznan University of Medical Science, Department Palliative Medicine, medical doctor

*Spain:*

Dr Xavier Gómez-Batist, MD- The ‘QUALY’ End of Life Catalan Oberservatory, medical doctor

Dr Sivia Paz Ruiz, The ‘QUALY’ End of Life Catalan Oberservatory, senior researcher

*UK:*

Prof. Sam Ahmedzai, MD- The University of Sheffield, Department Oncology, The Medical School, chair in palliative medicine

Bill Noble, MD- The University of Sheffield, Department Oncology, The Medical School, Medical doctor

Nisar Ahmed- Academic Unit of Supportive Care, School of Medicine and Biomedical Sciences, The University of Sheffield, researcher

Appendix C- Indicators set for the organisation of palliative care

**Table 3 Indicators set for the organisation of palliative care**

| **No** | **QI** | **Type** | **Application** | **Status** | **Database/  Hand search** | **Grey  literature** |
| --- | --- | --- | --- | --- | --- | --- |
| Definition of a palliative care service | | | | | | |
| 1 | All the services below are part of a comprehensive palliative care service:Palliaitve day care, Palliaitve home care support team, Hospice beds, Palliaitve hospital support team, Inpatiente palliative care hospital beds, Palliaitve care outpatient clinic, Bereavement support | Structure indicator | All settings | New developed |  |  |
| 2 | All the services below are part of a comprehensive palliative care service: Palliative day care | Structure indicator | All settings | New developed |  |  |
| 3 | All the services below are part of a comprehensive palliative care service: Palliative home care support team | Structure indicator | All settings | New developed |  |  |
| 4 | All the services below are part of a comprehensive palliative care service: Hospice beds | Structure indicator | All settings | New developed |  |  |
| 5 | All the services below are part of a comprehensive palliative care service: Palliative hospital support team | Structure indicator | All settings | New developed |  |  |
| 6 | All the services below are part of a comprehensive palliative care service: Inpatient palliative care hospital beds (e.g. palliative care unit) | Structure indicator | All settings | New developed |  |  |
| 7 | All the services below are part of a comprehensive palliative care service: Palliative care outpatient clinic | Structure indicator | All settings | New developed |  |  |
| 8 | All the services below are part of a comprehensive palliative care service: Bereavement support | Structure indicator | All settings | New developed |  |  |
| Access to palliative care | | | | | | |
| A: Access and availabilits (All settings) | | | | | | |
| 9 | A palliative care team is available at the request of the treating professional/team in all of the following settings: Day care, at home, Hospital, Hospice, Nursing home, Outpatient clinic, Day care | Process indicator | All settings | New developed |  |  |
| 10 | A palliative care team is available at the request of the treating professional/team in all of the following settings: Day care (excluding palliative day care) | Process indicator | All settings | New developed |  |  |
| 11 | A palliative care team is available at the request of the treating professional/team in all of the following settings: At home (or home replacing institution s.a mental institution, prison) | Process indicator | All settings | New developed |  |  |
| 12 | A palliative care team is available at the request of the treating professional/team in all of the following settings: Hospital | Process indicator | All settings | New developed |  |  |
| 13 | A palliative care team is available at the request of the treating professional/team in all of the following settings: Hospice | Process indicator | All settings | New developed |  |  |
| 14 | A palliative care team is available at the request of the treating professional/team in all of the following settings: Care home | Process indicator | All settings | New developed |  |  |
| 15 | A palliative care team is available at the request of the treating professional/team in all of the following settings: Outpatient clinic (excluding palliative care outpatient clinic) | Process indicator | All settings | New developed |  |  |
| 16 | For every professional/team specialised palliative care advice is available 24 hours a day, 7 days a week | Process indicator | All settings | Changed | [2-4] | [1] |
| 17 | Patients in need of palliative care and their families have access to palliative care facilities: Throughout the entire duration of their disease | Process indicator | All settings | Changed | [5,6] | [1] |
| 18 | Patients in need of palliative care and their families have access to palliative care facilities: With no extra financial consequences for the patient | Process indicator | All settings | Changed | [6,7] |  |
| 19 | Patients receiving palliative care have access to diagnostic investigations (e.g. X-rays, blood samples) regardless of their setting | Process indicator | All settings | Changed | [8] |  |
| Primary care (Home, Nursing home) | | | | | | |
| 20 | Palliative care is available for the patient and their family by:Phone | Process indicator | Primary care indicator | Changed | [2] | [1] |
| 21 | Palliative care is available for the patient and their family by:Visiting the patient | Process indicator | Primary care indicator | Changed | [2] | [1] |
| 22 | Palliative care is available for the patient and their family by:Bringing the patient to the service | Process indicator | Primary care indicator | Changed | [2] | [1] |
| 23 | For a palliative patient in a crisis , the following can be arranged within 24 hours: Admission | Process indicator | Primary care indicator | Changed | [9] | [1] |
| 24 | For a palliative patient in a crisis , the following can be arranged within 24 hours: An urgent discharge to patients home | Process indicator | Primary care indicator | Changed | [9] | [1] |
| 25 | For a palliative patient in a crisis , the following can be arranged within 24 hours: Transfer to another setting of care | Process indicator | Primary care indicator | Changed | [9] | [1] |
| B. Out of hours (All settings) | | | | | | |
| Staff | | | | | | |
| 26 | A member of a palliative care team is available 24 hours a day, 7 days a week: For palliative care consultation by phone | Process indicator | All settings | Changed | [2,3,10] | [1] |
| 27 | A member of a palliative care team is available 24 hours a day, 7 days a week: To provide bedside care in a crisis | Process indicator | All settings | Changed | [2,3,10] | [1] |
| Drugs | | | | | | |
| 28 | The following treatments are available for a palliative patient 24 hours a day, 7 days a week: Opioids and other controlled drugs | Structure indicator | Primary care indicator | Combined/ Changed | [11-14] | [1] |
| 29 | The following treatments are available for a palliative patient 24 hours a day, 7 days a week: Anticipatory medication for the dying patient | Structure indicator | Primary care indicator | Combined/ Changed | [11] | [1,13] |
| 30 | The following treatments are available for a palliative patient 24 hours a day, 7 days a week: Syringe drivers | Structure indicator | Primary care indicator | Combined/ Changed | [11] | [1,13] |
| C. Continuity of care (All settings) | | | | | | |
| 31 | There is a procedure for exchange of clinical information across caregivers, disciplines and settings | Process indicator | All settings | Changed | [7,8] |  |
| 32 | Before discharge/ transfer/ admission there is information transfer to the caregivers in the next setting regarding care and treatment | Process indicator | All settings | Changed |  |  |
| 33 | There is a professional caregiver per individual palliative patient nominated as responsible 'key worker' who coordinates care | Process indicator | All settings | Combined/ Changed | [15] | [1] |
| 34 | The responsible 'key worker' pays special attention to continuity of care within and across settings | Process indicator | All settings | Combined/ Changed | [15,16] | [1] |
| Inpatient setting (Hospital, Palliative care unit, Hospice) | | | | | | |
| 35 | General practitioners (GP's) are routinely called when a patient is being discharged home or transferred to another setting | Process indicator | Inpatient setting indicator | Changed | [3,9,17] |  |
| 36 | The discharge/ transfer letter of palliative care patients contains a multidimensional diagnosis, prognosis and treatment plan (see indicator 48 Clinical record ) | Structure indicator | Inpatient setting indicator | Changed | [18] |  |
| Primary care | | | | | | |
| 37 | The primary care out-of-hours service has handover forms (written or -electronic) with clinical information of all palliative care patients in the terminal phase at home | Structure indicator | Primary care indicator | Changed |  |  |
| Infrastructure | | | | | | |
| A. All settings | | | | | | |
| Infrastructure | | | | | | |
| 38 | Specialist equipment (e.g. anti decubitus mattresses, aspiration material, stoma care, oxygen delivery, special drug administration pumps, hospital beds, etc) is available for the nursing care of palliative care patients in each specific setting | Structure indicator | All settings | Changed |  | [1] |
| 39 | There is a dedicated room where multidisciplinary team meetings within one setting takes place | Structure indicator | All settings | New developed | [19,20] |  |
| 40 | There are dedicated facilities for multidisciplinary communications across settings: A dedicated room for meetings | Structure indicator | All settings | Changed | [20] |  |
| 41 | There are dedicated facilities for multidisciplinary communications across settings: Facilities for video or telephone conferences | Structure indicator | All settings | Changed | [20] |  |
| Information about care | | | | | | |
| 42 | There is an up to date directory of local caregivers and organisations that can have a role in palliative care | Structure indicator | All settings | New developed | [17] |  |
| 43 | There are dedicated information about the palliative care service: A website | Structure indicator | All settings | Changed | [7] |  |
| 44 | There are dedicated information about the palliative care service: Leaflets or brochures | Structure indicator | All settings | Changed | [7,19] |  |
| 45 | Patient information should be available in relevant foreign languages | Structure indicator | All settings | Changed | [3] |  |
| 46 | Appropriately trained translators should be available if professional caregivers and patient or family members do not speak the same language | Process indicator | All settings | Changed | [3] |  |
| 47 | There is a computerised medical record , to which all professional caregivers involved in the care of palliative care patients have access: Within one setting | Process indicator | All settings | Combined | [6,17] |  |
| It systems | | | | | | |
| 48 | There is a computerised medical record , to which all professional caregivers involved in the care of palliative care patients have access: Across different settings | Process indicator | All settings | Combined | [6,17] |  |
| B. Inpatient setting (Hospital, Palliative care unit, Hospice, Nursing home) | | | | | | |
| 49 | Consultations with the patient and/or family / informal caregivers are done in an environment where privacy is guaranteed (e.g. there is a dedicated room) | Structure indicator | Inpatient setting indicator | Changed | [12] |  |
| 50 | Dying patients are able to have a single bedroom if they want to | Process indicator | Inpatient setting indicator | New developed |  |  |
| 51 | There are facilities for a relative to stay overnight | Structure indicator | Inpatient setting indicator | New developed |  |  |
| 52 | Family members and friends are able to visit the dying patient without restrictions of visiting hours | Process indicator | Inpatient setting indicator | Changed | [12] |  |
| 53 | There is a private place (e.g. dedicated room) for saying goodbye to the deceased | Structure indicator | Inpatient setting indicator | New developed | [19] |  |
| C. Home care | | | | | | |
| 54 | For a palliative care patient staying at home there is the possibility, if needed, to provide someone (a volunteer or professional) to stay overnight if needed | Process indicator | Home care indicator | Changed | [19] |  |
| Assessment tools | | | | | | |
| 55 | There is a holistic assessment of palliative care needs of patients and their family caregivers (e.g. SPARC) | Process indicator | All settings | Changed | [9,21-26] |  |
| 56 | There is an assessment of pain and other symptoms using a validated instrument | Process indicator | All settings | Changed | [27-36] | [1,37] |
| Personnel palliative care services | | | | | | |
| A. Staff | | | | | | |
| 57 | The multidisciplinary team that provides palliative care consists of at least one of the following disciplines: Physician | Structure indicator | All settings | Changed | [8,39] | [38] |
| 58 | The multidisciplinary team that provides palliative care consists of at least one of the following disciplines:Nurse | Structure indicator | All settings | Changed | [8,39] | [38] |
| 59 | The multidisciplinary team that provides palliative care consists of at least one of the following disciplines:Spiritual/ religious caregiver | Structure indicator | All settings | Changed | [8,39] | [38] |
| 60 | The multidisciplinary team that provides palliative care consists of at least one of the following disciplines: Psychologist/Psychiatrist | Structure indicator | All settings | Changed | [8,39] | [38] |
| 61 | The multidisciplinary team that provides palliative care consists of at least one of the following disciplines: Social worker | Structure indicator | All settings | Changed | [8,39-40] | [38] |
| 62 | The multidisciplinary team that provides palliative care consists of at least one of the following disciplines: Physiotherapist | Structure indicator | All settings | Changed | [8,39] | [38] |
| 63 | The multidisciplinary team that provides palliative care consists of at least one of the following disciplines: Occupational therapist | Structure indicator | All settings | Changed | [8,39] | [38] |
| 64 | The multidisciplinary team that provides palliative care consists of at least one of the following disciplines: Dietitian | Structure indicator | All settings | Changed | [8,39] | [38] |
| 65 | The multidisciplinary team that provides palliative care consists of at least one of the following disciplines: Bereavement counselor | Structure indicator | All settings | Changed | [8,39] | [38] |
| 66 | The multidisciplinary team that provides palliative care consists of at least one of the following disciplines: Pharmacist | Structure indicator | All settings | Changed | [8,39] | [38] |
| 67 | New staff receives a standardised induction training | Process indicator | All settings | Changed |  | [41,1] |
| 68 | All team members have certified (accredited?) training in palliative care, appropriate to their discipline | Process indicator | All settings | Changed | [42] |  |
| 69 | All volunteers have training in palliative care. | Process indicator | All settings | Combined/ Changed |  | [13] |
| C. Support Systems | | | | | | |
| 70 | All team members have an annual appraisal | Process indicator | All settings | Changed |  | [41] |
| 71 | All team members who professionally deal with loss have access to a program for care for the carers | Process indicator | All settings | Changed | [23,24,43-45] |  |
| 72 | Satisfaction with working in the team is assessed (e.g. Team Climate Inventory) | Process indicator | All settings | Changed | [17,18,46-47] | [48] |
| D. Organisation of care | | | | | | |
| 73 | Palliative care services work in conjunction with the referring professional/ team | Process indicator | Inpatient setting indicator | New developed |  |  |
| 74 | There is a regular interdisciplinary/ multi-professional meeting to discuss palliative care patients: daily meetings to discuss day-to- day management of palliative care patients | Process indicator | All settings | Combined/ Changed | [44] |  |
| 75 | There is a regular interdisciplinary/ multi-professional meeting to discuss palliative care patients:weekly (inter- and multidisciplinary) meeting to review palliative care patients referrals and care plans | Process indicator | All settings | Combined/ Changed | [18,44,49,50] |  |
| E: Information sharing | | | | | | |
| 76 | All relevant team members are informed about patients who have died | Process indicator | Inpatient setting indicator | Changed |  | [41] |
| Documentation of clinical data | | | | | | |
| A: Clinical record (All settings) | | | | | | |
| 77 | For patients receiving palliative care a structured palliative care clinical record is used | Process indicator | All settings | Changed | [6,51] |  |
| 78 | The palliative care clinical record contains evidence of documentation of the following items: Clinical summary | Process indicator | All settings | Changed | [5] | [1] |
| 79 | The palliative care clinical record contains evidence of documentation of the following items: Physical aspects of care | Process indicator | All settings | Changed | [5,52] | [1] |
| 80 | The palliative care clinical record contains evidence of documentation of the following items: Psychological and psychiatric aspects of care | Process indicator | All settings | Changed | [5,12,39,53-55] | [1] |
| 81 | The palliative care clinical record contains evidence of documentation of the following items: Social aspects of care | Process indicator | All settings | Changed | [5,19,22,56,57] | [1] |
| 82 | The palliative care clinical record contains evidence of documentation of the following items:Spiritual, religious, existential aspects of care | Process indicator | All settings | Changed | [5,19,22] | [1] |
| 83 | The palliative care clinical record contains evidence of documentation of the following items: Cultural aspects of care | Process indicator | All settings | Changed | [5] | [1] |
| 84 | The palliative care clinical record contains evidence of documentation of the following items: Care of imminently dying patient | Process indicator | All settings | Changed | [5] | [1] |
| 85 | The palliative care clinical record contains evidence of documentation of the following items: Ethical, legal aspects of care | Process indicator | All settings | Changed | [5,12,19,58] | [1] |
| 86 | The palliative care clinical record contains evidence of documentation of the following items: Multidimensional treatment plan | Process indicator | All settings | Changed | [5,18,59,60] | [1] |
| 87 | The palliative care clinical record contains evidence of documentation of the following items: Follow up assessment | Process indicator | All settings | Changed | [5,16,61] | [1] |
| B. Timley documentation | | | | | | |
| Inpatiente setting (Hospital, Palliative care unit, Hospice, Nursing home) | | | | | | |
| 88 | Within 24 hours of admission there is documentation of the initial assessment of: Prognosis, Functional status, Pain and other symptoms, Psychosocial symptoms, The patient’s capacity to make decisions | Process indicator | Inpatient setting indicator | Changed | [12,18,46,62] |  |
| 89 | There is documentation that patients reporting pain or other symptoms at the time of admission, had their pain or other symptoms relieved or reduced to a level of their satisfaction within 48 hours of admission | Process indicator | Inpatient setting indicator | Changed | [18,46] |  |
| 90 | There is documentation about the discussion of patient preferences within 48 hours of admission | Process indicator | Inpatient setting indicator | Changed | [36] |  |
| 91 | A discharge/ transfer summary is available in the medical record within 48 hours after discharge/ transfer | Process indicator | Inpatient setting indicator | Changed | [36] |  |
| All settings | | | | | | |
| 92 | There is documentation of pain assessment at 4 hour intervals | Process indicator | All settings | Changed | [12,19,50,63] |  |
| 93 | The discussion of patient's preferences is reviewed on a regular basis (in parallel with disease progression) or on request of the patient | Process indicator | All settings | Changed | [64] |  |
| 94 | There is documentation that within 24 hours after patient transfer, the responsible physician in the receiving setting has visited the patient | Process indicator | All settings | Changed | [12,65] |  |
| 95 | There is documentation that within 24 hours after patient transfer, the new palliative care team in the receiving setting has visited the patient | Process indicator | All settings | Changed | [3] |  |
| Quality and safety issues | | | | | | |
| A: Quality policies | | | | | | |
| 96 | The palliative care service has a quality improvement program | Process indicator | All settings | Changed | [3,17,66] |  |
| 97 | There is documentation whether targets set for quality improvement have been met | Process indicator | All settings | Changed | [3] |  |
| 98 | Clinical audit are part of the quality improvement program | Process indicator | All settings | Changed | [3,67] | [48] |
| 99 | The setting uses a program about early initiation of palliative care (e.g. the Gold Standards Framework) | Process indicator | All settings | Changed |  | [1] |
| B. Adverse events | | | | | | |
| 100 | There is a register for adverse events | Process indicator | All settings | Changed | [5] | [48] |
| 101 | There is a documented procedure to analyse and follow up adverse events | Process indicator | All settings | Changed |  | [48] |
| C: Complaint procedure | | | | | | |
| 102 | There is a patient complaints procedure | Process indicator | All settings | Changed |  | [48,41] |
| Reporting clinical activity of palliative care services | | | | | | |
| 103 | The palliative care service uses a database for recording clinical activity | Process indicator | All settings | Changed | [68] | [69] |
| 104 | The following is part of the database: Diagnosis, Date of diagnosis, Date of referral, Date of admission to the palliative care service, Date of death, Place of death, Preferred place of death | Process indicator | All settings | Changed | [2,42,49-51,70-72] | [1] |
| 105 | From the database the service is able to derive: Time from diagnosis to referral to palliative care, Time from referral to initiation of palliative care, Time from initiation of palliative care to death, Frequency of unplanned consultations with the out-of-hours service for palliative care patients who are at home, Frequency of unplanned hospital admissions of palliative care patients, Percentage of non-oncological patients receiving palliative care | Process indicator | All settings | New developed | [73] |  |
| 106 | Based on the database, an annual report is made about the service | Process indicator | All settings | Changed | [17] | [4[1]] |
| Research | | | | | | |
| 107 | There is evidence that the palliative care service is involved in research in palliative care (e.g. authorship of publications, research grants) | Process indicator | All settings | Changed | [77] |  |
| Education | | | | | | |
| 108 | All health and social care students have standardised learning objectives for basic training in palliative care | Process indicator | All settings | Changed | [14,42,78,79] | [1] |
| 109 | All health and social care professionals have standardised learning objectives for continuing basic training in palliative care | Process indicator | All settings | New developed | [14,78,79] | [38] |
| 110 | There is a program for specialised training in palliative care for professionals working in a service that provides specialised palliative care | Process indicator | All settings | New developed |  | [38] |
